# Differential impacts of different keyboard inputting methods on reading and writing skills

**DOI:** 10.1038/s41598-018-35268-9

**Published:** 2018-11-21

**Authors:** Wai Ting Siok, Chun Yin Liu

**Affiliations:** Department of Linguistics, The University of Hong Kong, Pok Fu Lam Road, Hong Kong

## Abstract

Nowadays, typewriting has become an important mode of written communication. A report that typewriting may hinder Chinese children’s reading development has sparked substantial concern about whether typing on electronic devices would increase the rate of reading disorders, wherein children used a pronunciation-based input system that associates alphabet letters with phonemes in standard Chinese (Putonghua) and may conflict with the traditional visuomotor-based learning processes for written Chinese. If orthographic-based input methods that require good awareness of the orthographic structure of characters are used, different outcomes might be observed. This study examined the impact of participants’ experience in different typewriting methods on the literacy abilities of fluent Chinese-English bilingual readers. We found that orthographic-based typewriting measures correlated positively with Chinese reading measures, whereas pronunciation-based typewriting measures did not correlate with Chinese reading measures but correlated positively with English reading and spelling performance. Orthographic-based typewriters also performed better than pronunciation-based typewriters in Chinese reading and dictation when their age, typewriting skills and pre-University language ability were statistically controlled. Our findings based on two contrastive writing systems suggest that typewriting methods that tally with the learning principles of a writing system should be used to promote and preserve literacy skills in the digital era.

## Introduction

In the digital age, information is transmitted electronically, and communications are achieved digitally by texts, instant messages and video calls using various communication tools and social networking platforms. People at the present time seldom write by hand, as keyboarding techniques allow easy editing and saving of texts and offer useful and convenient tools such as spelling and grammar checks, text and stylistic formatting, translation, speech-text conversion, text sharing and simultaneous editing with others via the internet. In schools, electronic-based learning is promoted, and students are encouraged to use the internet to search for information and expand their knowledge. They are sometimes, or even often, required to complete assignments using computers and to submit them online or in printed form. Therefore, children nowadays are expected to have some basic typing or keyboarding skills when they start primary school, and handwriting is used less and less as a form of communication and literacy learning. This raises a key question: how do the increasingly used keyboarding techniques coupled with reduced handwriting affect people’s reading and writing skills?

Extensive experimental studies using alphabetic languages such as English have shown that phonological processing skills play a central role in reading acquisition, with impaired reading caused by phonological deficits^1–6^, but writing or hand-copying skills have not been found to predict reading outcomes^7–9^. Handwriting and typewriting in alphabetic languages entail similar perceptual and cognitive processes but different attentional and sensory-motor skills. Specifically, both writing modes start from either phonological (generated externally for dictation or internally for composition) or visual inputs (written or printed texts for copying or typewriting), and may involve processes such as speech or visual word recognition, idea formulation, semantic or lexical retrieval, phoneme-grapheme conversion (or spelling process), orthographic long-term and working memory^10^, visual analysis, graphic-motor planning and motoric hand movements^11–13^. Furthermore, given these two writing modes both require eye-hand coordination (or visual-motor integration), handwriting requires fine motor skills to trace and reproduce letter shape carefully, whereas typewriting requires searching and touching of the right key on a keyboard when letters are differentiated by the visuospatial location of the keys instead of the graphomotor demands. Handwriting may therefore offer an additional informative memory trace for letter-shape and visuomotor representation and lead to better letter representation and recognition^11,14–17^. Indeed, the neural substrates underlying letter recognition learned by handwriting and typewriting have been found to be different. Handwriting involves stronger activity in the left inferior frontal regions^18,19^, left anterior cingulate cortex^18^, bilateral inferior parietal lobule^19^, left posterior fusiform gyrus^18,20^ and dorsal precentral gyrus^20^, and in contrast typewriting involves additional or stronger brain activity in the left posteromedial intraparietal cortex and the more rostral part of the left premotor cortex^11^, and the right posterior fusiform gyrus^20^. The left Broca’s area is found to be responsible for, beyond verbal language functions, perceptual and motor sequencing^21^, the anterior cingulate cortex for conflict monitoring^22^, the bilateral inferior parietal lobule for hand–objects interactions^23,24^, and the left posterior fusiform and dorsal precentral gyrus for letter perception and letter writing^25^. The existence of dystypia (a lesion in the left frontal lobe that leads to typing impairment without writing or language disorders) further suggests that typewriting and handwriting are dissociated functions^26,27^.

These differences observed during the two writing processes have led to contrastive views on the impact of reduced handwriting on literacy development. Some argue that handwriting enables the establishment of more precise orthographic representation and accommodates an additional visual-motor memory of letter sequences and letter representation, which better enhances visual recognition of graphic shapes and letters than typewriting in children^17,28^ and adults^16,19^. It was reported that children receiving handwriting training had better spelling (but not reading) skills than those who received typewriting training^28^ (but a study^29^ found no significant difference between handwriting and typewriting skills in spelling acquisition). To the opposite, Masterson and Apel argued that the reported impact of handwriting and typewriting skills on spelling could have been caused by confounding factors such as insufficient proficiency in keyboard skills in some children^30^. When keyboard proficiency was included as a covariate, handwriting and typewriting training did not lead to differential spelling skills among grade 2 to 6 students. Similar findings were reported by a study^31^ which found an interaction between practice mode (handwriting or typewriting) and pre-existing keyboard skills in second graders.

In sum, compared with typewriting, handwriting may lead to better letter representation and recognition, yet its advantageous influence on spelling skills and its correlation with reading abilities have not been clearly established^7–9^. As handwriting and typewriting of an alphabetic script share similar perceptual and cognitive processes, increasing use of keyboarding techniques may cause less harm or even bring benefits to alphabetic learners. Young and disabled learners with motor immaturity or difficulties may be attracted to the colorful, interactive multimedia displays and encouraged to read more using digital devices, and the ease of typewriting will motivate them to write more; as such learners may otherwise be reluctant or even avoid reading and writing all together, any negative impact that typewriting may bring is outweighed by the possible advantages. Indeed, young and at-risk learners have been found to show faster literacy development by typewriting than handwriting training^32–36^, and a positive correlation between computer usage and letter knowledge was observed in pre-school learners^34^.

What will the impact of typewriting experience be on literacy development in the logographic Chinese system, a writing system fundamentally different from alphabetic ones? Written Chinese uses the character as a basic writing unit that is composed of unpronounceable strokes. Each character has a similar-sized square configuration irrespective of the number of strokes it contains, and maps onto a syllable and a morpheme without further decoding phonemes. The syllable-character mapping is not systematic and grapheme-phoneme correspondence rules that exist in all alphabetic languages are impossible in Chinese. Children need to be explicitly taught the pronunciation of each character and to rote memorize the stroke configuration and associate the visual form with its pronunciation and meaning. This visual-orthographic demand of written Chinese necessitates a prevalent teaching strategy: copy repeatedly the newly-instructed characters^37^. Through writing, children become aware of the orthographic structure of a character and learn to construct characters based on a unique pattern of strokes and components. This type of decoding is supposed to occur at the visual-orthographic level and will enhance children’s awareness of the character’s internal structure (a type of orthographic awareness). Hand copying skills and object drawing skills have been found to be in significant positive correlation with reading^37^ and writing skills^38^ in native Chinese speakers as well as Chinese-as-foreign-language learners^39^.

A pronunciation-based input method based on *Hanyu Pinyin* is the predominant Chinese input method in mainland China, which makes easy use of the QWERTY keyboard that was originally designed for alphabetic languages (the word QWERTY corresponds to the first six keys of the top letter row of a keyboard). Pinyin was adopted in the 1970s as a learning aid to help children and foreign learners pronounce modern Chinese characters. Pinyin is essentially a phonemic representation system, using the alphabet letters to represent sounds in standard Chinese, i.e., *Putonghua*. A similar system is used in Taiwan called *Zhu-Yin-Fu-Hao*. Once children have acquired the Pinyin principle, they are able to derive the sounds of characters through Pinyin printed above them and to match these sounds with the phonological codes that already exist in the mental lexicon. Thus, the Pinyin system links print with speech in Chinese and plays a role resembling the grapheme-phoneme correspondence rules in alphabetic languages. When people use the pinyin input method to type Chinese characters, phonological codes of characters (i.e., syllables) will be activated and converted into Pinyin phonetic pronunciation, and letters on the keyboard that correspond to the Pinyin symbols (ignoring tone marks) will be pressed. Thus, the production process relies on the pronunciation of words but not the visual-orthographic properties of characters that are indispensable to Chinese reading. As there are many homophones in Chinese, inputting the Pinyin string of a syllable (e.g.,/dong/) will retrieve a list of characters that share the same syllable (e.g., ) and the right character will need to be selected from these options. The list of characters is arranged based on frequency of use. The selection process will be speeded up if the full pinyin code of two-or-more-syllable words is typed (e.g., *dong-wu-yuan* for , meaning *zoo*), as “” will pop up as the first and only three-character-word choice, and only one selection instead of three (for each character) will be needed. The pinyin input method even allows users to type just the initials of the pinyin code of words (e.g., *d-w-y* in the previous example) to simplify the typing process. When *d-w-y* is typed, words that share the same syllable initials (e.g., *de-wei-yi*, —, *dong-wu-yuan* , *duo-wan-yuan* , ding-wei-yu , dan-wo-ye , dui-wo-you , to list only 6 choices) will be retrieved and arranged according to frequency of use. This feature helps people who are not familiar with the Pinyin codes to type more efficiently. Thus, although typing in pinyin may promote phonemic awareness and phonological memory^40^ and, henceforth, reading skills^41^, it conflicts with the traditional learning processes for written Chinese and may aversively affect children’s reading and, particularly, writing skills^42^.

Besides the Pinyin input method (or the Zhuyin method used in Taiwan), other keyboard input methods are used in Hong Kong and Taiwan, namely C*angjie*, *Quick* (or S*implified Cangjie*), *Stroke* and *Handwriting*. The first two are based on orthographic structure, the third on stroke composition of characters and the last one involves the actual handwriting process on a computer writing pad. The Cangjie and Quick input methods are built on 24 keys (A to Y excluding X) of the QWERTY keyboard. Each key is associated with a principle character and each principle character is associated with up to seven auxiliary semantic radicals or graphic components (for simplicity, we group semantic radicals and orthographic components together and call them *components* thereafter) that are variants of or visually similar to the principle character. For example, the key-P is associated with the principle character  (meaning *heart* or *mind*), which is associated with the seven auxiliary components  the left component of  and  without the upper horizontal stroke and the upper-right dot, which are either variants of or visually similar to the principal character . There are altogether 24 principal characters that are associated with about 100 auxiliary components. Components that cannot be classified into the 100 auxiliary components are grouped together into the “difficult” category and associated with the X-key (e.g., ). To type a character using the Cangjie input method, a character has to be decomposed into components, and up to five keys that correspond to the components are input in a pre-specified order (i.e., from top to bottom, from left to right, and from peripheral to center). This results in a unique Cangjie code for each character. To illustrate, the character  (meaning *flower*) is composed of the components  and . To type this character, we have to input the principle characters which correspond to these auxiliary components, namely  (T-key),  (O-key) and  (P-key), respectively, and then press the spacebar to confirm the input. The Quick method used the same key-codes as the Cangjie method, except that the former requires only the input of the first and final component codes to activate a list of characters that share the same first-final codes to let a typist to choose from. For the character , the first code  or T-key and the final code  or P-key are typed to pop up a list of characters that share the same first-final codes () and a typist needs to choose from among the list the right character s/he wants to type (the first choice in this example). Cangjie method is a faster method that requires good knowledge of how characters are decomposed into auxiliary components and how the 100 auxiliary components are associated with the 24 principle characters, while the Quick method is a slower method that is more typically used by novices. Typing in these methods may preserve the visuo-orthographic encoding of Chinese characters and thus have less negative impact on Chinese reading and writing skills. It is important to examine the impact of Chinese fluent readers’ experience with particular input methods on their reading and writing skills.

Here we intend to examine the correlational relationship between typewriting experience and reading and writing skills among people who have already achieved fluent reading and writing and have adequate typewriting skills. We studied college students not only because they are easy to recruit but also because they have to use more keyboarding techniques but less handwriting in their university life. If typewriting experience is found to be related to reading and writing performance in fluent readers, such relationship could be even more evident in developing children’s literacy skills and follow up studies on children should therefore be conducted. We anticipate that pronunciation-based (Pinyin) typewriting and orthographic-based typewriting may have different relationships with literacy skills. Participants’ Chinese reading, dictation, orthographic knowledge, typing speed and the time they spent on computer and smartphone typing and handwriting were assessed and compared between the two major typewriting methods (pronunciation-based vs orthographic-based). Correlational and regression analyses were then conducted to explore the relationship of different input methods with fluent readers’ reading and writing skills.

## Methods for Experiment 1

### Participants

Two hundred Chinese-English bilingual undergraduates at the University of Hong Kong (HKU) were recruited (56 males and 144 females, mean age = 20.4 ± 1.88 years). All of them were native Cantonese speakers and were highly proficient in English which they had acquired as a second language since the age of 3. Ninety-four participants used only orthographic-based input methods (Cangjie, Quick, Stroke or Handwriting) on their phone and computer, 97 used only pronunciation-based input methods (Pinyin or Jyut-ping, a Cantonese-based pronunciation-based input method), while 9 used both methods. They received monetary reward for their participation and gave informed consent prior to experiment. The study was approved by the Human Research Ethics Committee of the University of Hong Kong. The methods were carried out in accordance with the approved guidelines.

### Test Materials and Procedures

Before the experimental tasks, participants needed to fill out a questionnaire asking for their demographic information, public examination results of Chinese language (i.e., the Hong Kong Diploma of Secondary Education Examination, or HKDSE), their usual input methods for smartphones and computers, the time they spent on typing Chinese on smartphones and computers daily and the time they spent on Chinese handwriting weekly. They then participated in four behavioral tasks in the following sequence: the dictation task, the typewriting speed task, the character reading task and the orthographic task. Because there is no standardized reading and writing ability test for adults in Hong Kong or China, we constructed the reading, dictation and orthographic tasks following the common practice in Chinese reading research^37,43^. The questionnaire and the behavioral tasks were administered individually within a 30-minute session.

We used the HKDSE results as a validity benchmark for our reading-related tasks. The HKDSE is an achievement examination administered by the Hong Kong Examination and Assessment Authority and serves as an entrance examination to higher education institutions in Hong Kong, with Chinese Language, English Language, Mathematics and Liberal Studies as the four core subjects. The HKDSE Chinese language exam adopts a criterion-referenced standards-based assessment method which contains five subtests assessing students’ reading, writing, speaking, listening and integrated skills (a combination of reading and listening skills). Candidates’ assessment results in each subtest as well as their overall performance in this subject are reported against a set of standards divided into 5 levels (levels 1 to 5), with 1 being the lowest. Within level 5, the best performers are awarded a 5** (equivalent to a score of 7) and the next best performers are awarded 5* (equivalent to a score of 6).

### Dictation task

Participants were instructed to listen to and write down 48 disyllabic words (a total of 96 characters) on a piece of paper, as each word being presented with an 8-second interval. The printed frequency of the 96 characters ranged from 1 to 2950 in about 662,000 (mean = 148.8, SD = 430.1)^44^. The number of strokes ranged from 4 to 25 (mean = 12.8, SD = 4.12). The disyllabic words (e.g., , meaning *noble*) are used quite frequently in oral discourse but contain a character with low printed frequency (e.g., the printed frequency of  is 9). To standardize the presentation of word stimuli, the words were recorded by a native male Cantonese speaker and audio recording was presented during the experiment. The task lasted for 7 minutes and 20 seconds. Each correct character scored 1 point and the highest score was 96. The Cronbach’s Alpha reliability coefficient (a statistical index of internal consistency) for this test is 0.92.

### Orthographic knowledge task

Participants were instructed to proofread 60 disyllabic word stimuli printed on a piece of paper; the stimuli consisted of 32 real words (e.g., , meaning *in a critical condition*) and 28 illegal compounds. Each illegal compound was formed by replacing a character in a real two-character word with either a (i) homophonic but visually-dissimilar character (e.g.,  is an illegal compound in which  is homophonic to  and  is the real compound meaning *wishful thinking*; there were 11 such trials), (ii) visually-similar but non-homophonic character (e.g.,  is an incorrect compound in which  is visually similar but phonologically different from  and  is the correct compound meaning *obstruct*; there were 8 such trials), or (iii) homophonic and visually similar character (e.g.,  is the resultant illegal compound in which  is homophonic and visually similar to  and  is the correct compound meaning *wall*; there were 9 such trials). Participants were asked to correct the illegal compounds by changing the incorrect characters and to ignore the legal compounds. They were given 5 minutes to complete the task. If an incorrect character in the illegal compounds was correctly changed or if the participant did not make any change to a legal compound, one mark was rewarded. The highest score was 60. The Cronbach’s Alpha reliability coefficient for this test is 0.73.

### Character reading task

Participants were instructed to read aloud 300 characters printed on a page as fast and accurately as possible. They were told to either skip or guess the pronunciation of a character if they did not know the correct pronunciation of that character. The printed frequency of the 300 characters ranged from 1 to 107 (in 662,000)^44^ and the stroke number of the characters ranged from 4 to 28 (mean = 12.46, SD = 4.18). Each correct item had 1 score and the highest score was 300. The Cronbach’s Alpha reliability coefficient for this test is 0.91.

### Typewriting Speed

Participants’ typewriting speed on smartphone and computer was measured separately using their usual input method. Two different news passages, one had 200 characters and the other 213, were prepared and each participant typed one passage using smartphone and another using computer. The two passages were pseudo-randomly assigned to either smartphone or computer typing such that each passage was typed by an equal number of participants on each device. Participants were asked to type as fast and accurately as possible within 2 minutes for each electronic mode. They were instructed to skip punctuation marks and characters that they did not know the input code. An iPhone 4, using the QWERTY layout, was used for the smartphone typing session, and participants’ own laptop computer (for those who used IOS) or the experimenter’s computer (for those who used Windows) was used for the computer typing session. Each correctly typed character had 1 score. A typewriting speed score was calculated by adding the number of words correctly typed for each electronic mode within 2 minutes.

## Results and Discussion for Experiment 1

Demographic information and descriptive statistics including means and standard deviations for all participants on each test are presented in Table 1. We excluded the 9 participants who used both orthographic-based and pronunciation-based inputting methods from our statistical analysis, leaving 191 participants available for the analysis. For participants who used orthographic-based inputting methods, 72 used the Quick method, 17 used Cangjie, 3 used a Stroke-based method and 2 used handwriting input. Since the result patterns did not differ if we included all the 94 participants or just the 72 who used Quick, we collapsed data of participants using these four typewriting methods together to increase statistical power and for parsimony in data analysis and presentation.

**Table 1:** Demographic information and descriptive statistics including means and standard deviations for all participants on each test.

	All	Orthographic-based input method (1)	Pronunciation-based input method (2)	Independent sample t-test (p < 0.05)
Number of participants	191	94	97	
Mean age	20.39 (1.91)	20.71 (2.26)	20.07 (1.44)	(1) > (2)
Gender	F:137 M:54	F: 66 M:28	F:71 M:26	
Year of study at university	2.67 (1.18)	2.98 (1.15)	2.37 (1.15)	(1) > (2)
DSE Chinese Overall	4.85 (1.20)	4.77 (1.21)	4.94 (1.19)	
DSE Chinese Reading	4.88 (1.52)	4.78 (1.48)	4.98 (1.55)	
DSE Chinese Writing	4.63 (1.61)	4.55 (1.67)	4.70 (1.56)	
DSE Chinese Listening	4.90 (1.51)	4.64 (1.47)	5.16 (1.52)	(1) < (2)
DSE Chinese Speaking	4.47 (1.56)	4.51 (1.63)	4.43 (1.50)	
DSE Chinese Integrated	5.48 (1.34)	5.44 (1.36)	5.53 (1.33)	
Dictation (MAX = 96)	64.17 (12.44)	65.78 (11.89)	62.62 (12.81)	
Character reading (MAX = 300)	267.87 (15.15)	269.18 (16.38)	266.60 (13.82)	
Orthographic task (MAX = 60)	43.45 (18.87)	43.31 (4.31)	43.59 (5.31)	
Typing speed (smartphone, # words correctly typed)	60.26 (36.79)	55.36 (13.18)	65.01 (22.14)	(1) < (2)
Typing speed (computer, # words correctly typed)	65.27 (50.16)	53.78 (33.07)	76.40 (36.95)	(1) < (2)
Overall typing speed	125.53 (41.54)	109.14 (40.36)	141.41 (53.70)	(1) < (2)
Daily typing time on smartphones (in min)	66.68 (36.45)	62.87 (41.35)	70.36 (41.61)	(1) < (2)
Daily typing time on computers (in min)	49.08 (72.42)	43.09 (36.23)	54.90 (35.90)	(1) < (2)
Total typing time	115.76 (36.72)	105.96 (72.92)	125.26 (71.02)	
Weekly time on hand writing (in min)	40.45 (37.72)	39.57 (37.06)	41.29 (36.55)	

Because the two groups differed in some demographic variables that may affect reading and writing performance (see Table 1), we conducted one-way analysis of covariance (ANCOVA) to compare the group differences in reading and dictation while statistically controlling for differences in age, HKDSE Chinese overall performance, typewriting speed and total time spent on typewriting. We included HKDSE Chinese overall performance as a covariate (or an autoregressor) because participants’ current reading and writing performance would be affected by their prior performance (as measured by HKDSE Chinese overall performance). Results of ANCOVA revealed that the two groups differed significantly in Chinese Dictation [adjusted means for the orthographic and pronunciation groups are 66.95 and 61.48, respectively; *F*(1,185) = 9.17, *p* < 0.005, Eta squared, η^2^ = 0.04] and Chinese Character Reading [adjusted means for the orthographic and pronunciation groups are 270.09 and 265.72, respectively; *F*(1,185) = 3.92, *p* < 0.05, η^2^ = 0.02]. These findings show that students using pronunciation-based typewriting had a lower dictation and reading score in Chinese when compared with those using orthographic-based typewriting.

Pearson correlation coefficients among the behavioral measures were presented in Table 2 for all participants, Table 3 for the orthographic-based typewriting group (simply termed as the “orthographic group”), and Table 4 for the pronunciation-based typewriting group (simply termed as the “pronunciation group”). We used the statistical software COCOR^45^ to compare between two correlations, and reported Pearson and Filon’s z for within-group comparisons and Fisher’s z for between-group comparisons. For all participants (n = 191), HKDSE Chinese correlated moderately with dictation, character reading, and orthographic knowledge (*r* = 0.29, *p* < 0.001, *r* = 0.34, *p* < 0.001, and *r* = 0.24, *p* < 0.001, respectively) (see Table 2). This suggests that our character or word level measures could reflect to some extent a person’s overall Chinese language performance. Character reading correlated significantly with dictation (*r* = 0.60, *p* < 0.001), and both character reading and dictation correlated significantly with orthographic knowledge (*r* = 0.53, *p* < 0.001 and *r* = 0.73, *p* < 0.001, respectively), with the latter correlation found to be significantly larger than the former (Pearson and Filon’s z = 4.15, p < 0.0005, two-tailed). Thus, good reading and dictation skills are both contingent on good orthographic knowledge in Chinese, with dictation skills requiring better orthographic knowledge than reading.

**Table 2:** Pearson correlation coefficients between various behavioral measures for all participants (n = 191).

	1	2	3	4	5	6
1 Dictation	—					
2 Character reading	0.60***	—				
3 Orthographic knowledge	0.73***	0.53***	—			
4 Time on typewriting	0.15*	0.09	0.16*	—		
5 Time on handwriting	0.15*	0.07	0.06	0.29***	—	
6 Typewriting speed	0.17*	0.13ǂ	0.22**	0.41***	0.12^ǂ^	—
7 DSE Chinese	0.29***	0.34***	0.24***	0.17*	0.07	0.12^ǂ^

^ǂ^p < 0.1. ^*^p < 0.05. ^**^p < 0.005. ^***^p < 0.001.

**Table 3:** Pearson correlation coefficients between various behavioral measures for the orthographic-based typewriting group (n = 94).

	1	2	3	4	5	6
1 Dictation	—					
2 Character reading	0.68***	—				
3 Orthographic knowledge	0.71***	0.62***	—			
4 Time on typewriting	0.25*	0.15	0.18^ǂ^	—		
5 Time on handwriting	0.17	0.07	0.09	0.34***	—	
6 Typewriting speed	0.32**	0.17	0.34***	0.38***	0.20^ǂ^	—
7 DSE Chinese	0.31**	0.43***	0.20*	0.08	0.03	−0.01

^ǂ^p < 0.1. *p < 0.05.**p < 0.005. ***p < 0.001.

**Table 4:** Pearson correlation coefficients between various behavioral measures for the pronunciation-based typewriting group (n = 97).

	1	2	3	4	5	6
1 Dictation	–					
2 Character reading	0.51***	—				
3 Orthographic knowledge	0.77***	0.46***	—			
4 Time on typewriting	0.10	0.06	0.13	—		
5 Time on handwriting	0.14	0.07	0.03	0.24*	—	
6 Typewriting speed	0.16	0.18^ǂ^	0.15	0.39***	0.07	—
7 DSE Chinese	0.30**	0.25*	0.28*	0.26*	0.11	0.20^ǂ^

^ǂ^p < 0.1. *p < 0.05.**p < 0.005. ***p < 0.001.

For the orthographic group, typewriting speed and time correlated significantly with dictation (*r* = 0.32, *p* < 0.005 and *r* = 0.25, *p* < 0.05, respectively) but not with character reading. This suggests that orthographic-based typewriting skills have closer relationship with dictation but not with character reading, and the correlation between typewriting speed and dictation (*r = *0.32) was stronger than that with reading (*r* = 0.17) (Pearson and Filon’s *z* = 1.89, *p* < 0.05, one-tailed). Orthographic knowledge correlated significantly with typewriting speed (*r* = 0.34, *p* < 0.001) and marginally with typewriting time (*r* = 0.18, *p* = 0.077), implying either that good orthographic-based typewriting skill needs good orthographic knowledge and more practice in typewriting, or that orthographic-based typewriting may promote orthographic knowledge. For the pronunciation group (n = 97), typewriting speed and time did not significantly correlate with character reading, dictation and orthographic knowledge (all *p*s > 0.05), suggesting that this typewriting method does not incorporate the cognitive skills that are crucial for Chinese reading and writing.

To find out which cognitive factors predict character reading and dictation in each typewriting group, we conducted a series of fixed-order hierarchical multiple regressions, with character reading and dictation as two separate criterion variables. Age and HKDSE Chinese entered the regression equations as Steps 1 and 2, respectively, to control for variances of past reading experience and performance. As the medium of instruction at HKU is English (except in the School of Chinese) and the use of electronic devices in the classroom has become the norm on university campus, most undergraduates no longer read Chinese academic texts and handwrite less for academic and communicative purposes. Therefore, HKDSE Chinese results would reflect the pre-university language skills, while the character reading and dictation tests would reflect the post-university (or post-typewriting) literacy skills. Previous studies have found that hand copying skills positively correlated with reading^30^ and writing skills^31^ in native Chinese speakers. We suspected that time spent on handwriting might differentially modulate the impact of pronunciation-based and orthographic-based typewriting experience on Chinese reading and dictation. We thus entered time spent on handwriting as Step 3 in the regression analyses. Time spent on typewriting, typewriting speed and orthographic knowledge were entered separately as Step 4 in the regression analyses (see Table 5 for the regression table).

**Table 5:** Summary of hierarchical multiple regressions that tested the predictive power of orthographic knowledge and typewriting measures on Chinese reading and dictation after controlling for differences in age, DSE Chinese and handwriting time.

Step	Variable	Orthographic-based typewriting group						Pronunciation-based typewriting group					
		Character reading		Dictation		Typewriting Speed		Character reading		Dictation		Typewriting Speed	
		ΔR^2^	β	ΔR^2^	β	ΔR^2^	β	ΔR^2^	β	ΔR^2^	β	ΔR^2^	β
1	Age	0.000		0.016		0.000		0.008		0.000		0.015	
2	HKDSE Chinese	0.195^***^		0.085**		0.000		0.057*		0.089**		0.032^ǂ^	
3	Handwriting time	0.005		0.024		0.040^ǂ^		0.003		0.011		0.004	
4	Typewriting speed	0.027^ǂ^	0.169^ǂ^	0.087**	0.301**	—	—	0.016	0.131	0.000	0.000	—	—
4	Typewriting time	0.011	0.114	0.034^ǂ^	0.197^ǂ^	0.112^***^	0.357^***^	0.000	−0.015	0.009	0.099	0.132^***^	0.386^***^
4	Orthographic knowledge	0.293^***^	0.555^***^	0.428^***^	0.671^***^	0.114^***^	0.346^***^	0.164^***^	0.424^***^	0.518^***^	0.753^***^	0.008	0.091

^ǂ^p < 0.10. ^*^p < 0.05. ^**^p < 0.005, ^***^p < 0.001.

Orthographic knowledge was found to be the most significant predictor of character reading [ΔR^2^ = 0.29, ΔF(1,89) = 51.30, p < 0.001 for the orthographic group and ΔR^2^ = 0.16, ΔF(1,92) = 19.64, p < 0.001 for the pronunciation group] and dictation [ΔR^2^ = 0.43, ΔF(1,89) = 85.01, p < 0.001 for the orthographic group and ΔR^2^ = 0.52, ΔF(1,92) = 124.79, p < 0.001 for the pronunciation group]. Typewriting speed was predictive of dictation [ΔR^2^ = 0.09, ΔF(1,89) = 9.85, p < 0.005] in the orthographic group, but no typewriting measures were found to be predictive of reading and dictation in the pronunciation group. When typewriting speed served as the criterion variable in the hierarchical regression analysis, orthographic knowledge accounted for significant portion of variance in typewriting speed for the orthographic group [ΔR^2^ = 0.11, ΔF(1,89) = 11.95, p < 0.001] but not for the pronunciation group [ΔR^2^ = 0.01, ΔF(1,92) = 0.74, p = 0.393] (see Table 5). These findings indicate that orthographic knowledge is needed for good orthographic-based typewriting skills, although cause-effect relationship between these two variables could not be established because of the cross-sectional nature of the data. Such relationship is not observed in the pronunciation group, implying that pronunciation-based typewriting does not require orthographic knowledge. It is also likely that pronunciation-based input methods do not promote orthographic knowledge, a skill that is crucial for good Chinese reading and dictation performance, and thus the long-term reliance on it may negatively impact Chinese reading and writing ability.

It is interesting to note that time spent on handwriting did not correlate significantly with either reading or dictation performance in either group, possibly because the participants of this study were highly academic-achieving, proficient Chinese readers whose literacy skills were less vulnerable to reduced handwriting practice. Importantly, their reading and dictation skills were, to some extent, affected by their keyboarding practice. Those who used more and were good at orthographic-based typewriting methods had better reading and dictation skills than those who used pronunciation-based typewriting methods. Orthographic-based, but not pronunciation-based, input methods may enhance or preserve orthographic knowledge, as typing in orthographic-based methods require explicit awareness of orthographic structure of characters. Thus, people who have better orthographic knowledge type faster, which in turn helps refreshing their orthographic knowledge as well as reading and writing skills. These findings together may imply that typewriting methods involving similar cognitive processes crucial for successful reading acquisition will preserve, if not enhance, reading skills of fluent readers. To testify this hypothesis, we conducted Experiment 2, an English version of Experiment 1, on a smaller sample of the Chinese-English bilingual participants (n = 140). An oddity test that measures participants’ phonological awareness was also included to examine its relationship with reading and typing measures. As was reviewed above, typewriting and handwriting in alphabetic systems involve similar cognitive processes. We predict that English typewriting experience positively correlates with English reading and spelling performance.

## Methods for Experiment 2

### Participants

The 191 participants of Experiment 1 were invited to return for the English experiment and 140 of them participated in Experiment 2 (42 males and 98 females, mean age = 20.4 ± 1.41 years) (see Table 6). Sixty participants used orthographic-based input methods for Chinese typewriting, and 80 used pronunciation-based input methods. They received monetary reward for their participation and gave informed consent prior to experiment. The experiment was  approved by the Human Research Ethics Committee of the University of Hong Kong. The methods were carried out in accordance with the approved guidelines.

**Table 6:** Demographic information and descriptive statistics including means and standard deviations for all participants on each test in Experiment 2.

	All	Orthographic-based input method (1)	Pronunciation-based input method (2)	Independent-Sample T-test (p < 0.05)
Number of participants	140	60	80	
Mean age	20.36 (1.41)	20.70 (1.25)	20.11 (1.47)	(1) > (2)
Gender	F: 98 M:42	F:43 M:17	F:55 M:25	
Year of study at university	2.71 (1.20)	3.08 (1.14)	2.44 (1.18)	(1) > (2)
DSE English Overall (1-7, 7 being the best)	5.16 (1.06)	5.15 (1.06)	5.18 (1.08)	
DSE English Reading	5.42 (1.22)	5.43 (1.13)	5.41 (1.30)	
DSE English Writing	5.04 (1.31)	5.18 (1.32)	4.93 (1.30)	
DSE English Listening	5.11 (1.35)	5.15 (1.25)	5.08 (1.42)	
DSE English Speaking	5.30 (1.25)	5.20 (1.20)	5.38 (1.30)	
English Word reading (MAX = 300)	284.91 (14.27)	282.45 (16.61)	286.76 (12.01)	
WJ Word Identification (MAX = 76)	66.84 (4.43)	66.12 (4.88)	67.38 (4.02)	
WJ Word Attack (MAX = 28)	24.84 (2.93)	24.25 (3.34)	25.28 (2.51)	(1) < (2)
English Dictation/Spelling (MAX = 96)	63.46 (11.78)	62.13 (11.47)	64.45 (12.00)	
English Orthographic task (MAX = 60)	42.64 (5.86)	41.87(5.49)	43.23 (6.09)	
Phonological awareness (MAX = 20)	14.51 (3.59)	13.83 (3.59)	15.01 (3.52)	
Typing speed in English (smartphone, # words correctly typed)	57.86 (11.78)	59.75 (13.85)	56.44 (9.80)	
Typing speed in English (computer, # words correctly typed)	75.38 (21.76)	78.27 (21.30)	73.21 (21.98)	
Daily typing time in English on smartphones (in min)	76.07 (39.65)	74.00 (42.77)	77.63 (37.34)	
Daily typing time in English on computers (in min)	75.64 (38.16)	72.00 (40.10)	78.38 (36.66)	
Daily time on English hand writing (in min)	32.79 (30.94)	29.50 (26.20)	35.25 (34.01)	
DSE Chinese Overall (1-7, 7 being the best)	4.85 (1.19)	4.87 (1.14)	4.84 (1.23)	
DSE Chinese Reading	4.90 (1.57)	4.83 (1.49)	4.95 (1.64)	
DSE Chinese Writing	4.67 (1.58)	4.82 (1.64)	4.56 (1.54)	
DSE Chinese Listening	4.96 (1.44)	4.80 (1.29)	5.09 (1.54)	
DSE Chinese Speaking	4.41 (1.58)	4.43 (1.60)	4.39 (1.58)	
DSE Chinese Integrated	5.48 (1.33)	5.52 (1.26)	5.45 (1.40)	
Chinese Dictation (MAX = 96)	63.97 (12.71)	66.15 (11.64)	62.34 (13.29)	
Chinese Word reading (MAX = 300)	267.55 (14.98)	269.82 (15.08)	265.85 (14.76)	
Chinese Word per minute (CWPM)	57.17 (12.38)	58.19 (12.08)	56.40 (12.62)	
Chinese Orthographic task (MAX = 60)	43.71 (4.71)	43.78 (4.03)	43.66 (5.19)	
Typing speed in Chinese (smartphone, # words correctly typed)	60.77 (17.76)	56.75 (12.69)	63.79 (20.32)	(1) < (2)
Typing speed in Chinese (computer, # words correctly typed)	67.55 (35.72)	57.92 (32.22)	74.78 (36.70)	(1) < (2)
Overall typing speed	128.32 (47.34)	114.67 (38.14)	138.56 (51.08)	(1) < (2)
Daily typing time in Chinese on smartphones (in min)	68.57 (41.62)	69.50 (42.60)	67.88 (41.13)	
Daily typing time in Chinese on computers (in min)	48.43 (35.64)	46.00 (38.65)	50.25 (33.34)	
Total typing time	117.00 (71.18)	115.50 (76.30)	118.13 (67.56)	
Weekly time hand writing in Chinese (in min)	40.29 (36.57)	43.50 (39.22)	37.88 (34.50)	

### Test Materials and Procedures

Before the experimental tasks, participants needed to fill out a questionnaire asking for their demographic information, public examination results of the HKDSE English subject, the time they spent on English typewriting on smartphones and computers daily, and the time they spent on English handwriting weekly. They then participated in five behavioral tasks in the following sequence: the dictation task, the typewriting speed task, the phonological awareness task, the orthographic task, and three word reading tasks: a self-constructed English word reading task, and the Woodcock-Johnson III Letter-Word Identification (WJ-WID) and Word Attack (WJ-WA) subtests^46^. The questionnaire and the behavioral tasks were administered individually within a 35-minute session.

### Spelling/Dictation task

Participants were instructed to listen to and write down 96 English words, each being presented with a 4-second interval. The printed frequency of the 96 words ranged from 0 to 783 in one million (mean = 58.61, SD = 113.39)^47^. To standardize the presentation of word stimuli, the words were recorded by a native female English speaker and the audio recording was presented during the experiment. The task lasted for 7 minutes and 50 seconds. Each correct word scored 1 point and the highest score was 96. The Cronbach’s Alpha reliability coefficient for this test is 0.92.

### Orthographic knowledge task

Participants were instructed to proofread 60 short English phrases. Thirty-one of the phrases were correct while the remaining 29 contained an incorrect word. Each of the correct short phrase contained a word that had homophonic neighbors (e.g., a *waste* of time, in which *waste* is a homophone of *waist*). Among the incorrect words, 9 were homophones (e.g. the basic *principal* in science – the correct word should be *principle*), 10 were misspelt words (e.g. two *seperate* issues – the correct word should be *separate*) and 10 were similarly spelt words but non-homophones (e.g. an obtuse *angel* —the correct word should be *angle*). The participants were instructed to circle the incorrect word and write down the correct word next to it. Participants were given 5 minutes to complete the task. Each correct item had 1 score and the highest score was 60. The Cronbach’s Alpha reliability coefficient for this test is 0.78.

### English word reading tasks

We used the following three tasks to assess participants’ English reading proficiency:

#### English word reading

Participants were instructed to read aloud 300 words printed on an A4 card as fast and accurately as possible. They were told to either skip or guess the pronunciation of a word if they did not know the correct pronunciation of that word. The words had frequency ranged from 2 to 279 (mean = 40.68, SD = 43.48) (in one million)^47^ and word length ranged from 2 to 7 letters (mean = 6.78, SD = 2.24). Each correct item had 1 score and the highest score was 300. The Cronbach’s Alpha reliability coefficient for this test is 0.94.

#### Woodcock Johnson III Subtest: Letter-Word Identification (WJ-WID)

The WJ-WID^46^ assesses the ability to decode real words. The word list is composed of 13 English letters and 63 real words. Words are ordered according to difficulty. The procedure was identical to the previous word reading task. Each correct item had 1 score and the highest score was 76. The Cronbach’s Alpha reliability coefficient for this test is 0.80.

#### Woodcock Johnson III Subtest: Word Attack (WJ-WA)

The WJ-WA assesses the ability to decode pseudowords and knowledge in grapheme-phoneme correspondence. The stimulus list is composed of 5 English letters and 23 pseudowords. They are ordered according to difficulty. The procedure was identical to the previous word reading task. Each correct item had 1 score and the highest score was 28. The Cronbach’s Alpha reliability coefficient for this test is 0.80.

### Typewriting Speed

Participants’ English typewriting speed on smartphone and computer was measured separately. Two different news passages, one had 328 words and the other 338, were prepared and each participant typed one passage using smartphone and another using computer. The two passages were pseudo-randomly assigned to either smartphone or computer typing so that each passage was typed by an equal number of participants on each device. Participants were asked to type as fast and accurately as possible within 2 minutes for each electronic mode. They were instructed to skip punctuation marks on smartphone but type everything on computer. An iPhone 4, using the QWERTY layout, was used for the smartphone typing session, and the experimenter’s computer was used for the computer typing session. Each correctly typed character had 1 score.

### Phonological awareness

Participants’ phonological awareness was measured by an oddity task. They were presented with twenty sets of four words. For each set, one of the words has a different onset or rime from the other three words, with ten sets having different onsets (e.g., *ben*, *put*, *people*, *pack*; *ben* is the odd one with a different beginning phoneme), and the other ten different rimes (e.g., *chair*, *share*, *tier*, *wear*; *tier* is the odd one with a different rime). Participants were instructed to identify the word that had a different onset or rime. Each correct item had 1 score and the highest score was 20. The Cronbach’s Alpha reliability coefficient for this test is 0.77.

## Results and Discussion for Experiment 2

Demographic information and descriptive statistics including means and standard deviations for all participants on each test are presented in Table 6. Pearson correlation coefficients among the behavioral measures were presented in Table 7 for all participants, Table 8 for the orthographic group, and Table 9 for the pronunciation group. Similar to the Chinese study, HKDSE English correlated significantly with English word reading, Woodcock Johnson word identification (WJ-WID), Woodcock Johnson word attack (WJ-WA), dictation, orthographic knowledge and phonological awareness (*r* = 0.61, *p* < 0.001, *r* = 0.55, *p* < 0.001, *r* = 0.54, *p* < 0.001, *r* = 0.67, *p* < 0.001, *r* = 0.58, *p* < 0.001, and *r* = 0.47 *p* < 0.001, respectively), suggesting that our self-constructed measures could reflect quite well a person’s overall English language performance. WJ-WID and WJ-WA correlated significantly with our English word reading (*r* = 0.85, *p* < 0.001 and *r* = 0.75, *p* < 0.001, respectively) and dictation tests (*r* = 0.71, *p* < 0.001 and *r* = 0.65, *p* < 0.001, respectively), further indicating that these self-constructed English tests have good validity in measuring one’s English performance. Orthographic knowledge correlated significantly with word reading (*r* = 0.67, *p* < 0.001), WJ-WID (*r* = 0.64, *p* < 0.001), WJ-WA (*r* = 0.50, *p* < 0.001), dictation (*r* = 0.73, *p* < 0.001), and phonological awareness (*r* = 0.53, *p* < 0.001), whereas phonological awareness correlated significantly with word reading (*r* = 0.57, *p* < 0.001), WJ-WID (*r* = 0.52, *p* < 0.001), WJ-WA (*r* = 0.55, *p* < 0.001), and dictation (*r* = 0.53, *p* < 0.001). Thus, orthographic knowledge and phonological awareness are important to English word reading and dictation in English-as-second-language (ESL) speakers, with the impact of orthographic (or spelling) knowledge on reading and dictation found to be significantly stronger than that of phonological awareness (Pearson and Filon’s z = 1.78, p < 0.05, one-tailed, for word reading and z = 3.35, p < 0.001, two-tailed, for dictation).

**Table 7:** Pearson correlation coefficients between various behavioral measures in English for all participants (n = 140). WJ-WID: Woodcock-Johnson word identification. WJ-WA: Woodcock-Johnson word attack.

	1	2	3	4	5	6	7	8	9
1 English Dictation	—								
2 English Word Reading	0.754***	—							
3 WJ-WID	0.714***	0.851***	—						
4 WJ-WA	0.648***	0.748***	0.695***	—					
5 Orthographic knowledge	0.726***	0.674***	0.640***	0.497***	—				
6 Phonological awareness	0.511***	0.538***	0.507***	0.531***	0.505***	—			
7 English handwriting time	−0.043	0.039	−0.030	−0.039	0.096	0.019	—		
8 English typewriting time	0.282***	0.293***	0.174*	0.156^ǂ^	0.290***	0.152^ǂ^	0.289***	—	
9 English typewriting speed	0.567***	0.459***	0.392***	0.373***	0.463***	0.275***	−0.072	0.335***	—
10 HKDSE English	0.671***	0.609***	0.546***	0.535***	0.582***	0.429***	−0.004	0.108	0.433***

^ǂ^p < 0.1. ^*^p < 0.05. ^**^p < 0.005. ^***^p < 0.001.

**Table 8:** Pearson correlation coefficients between various behavioral measures in English for the orthographic-based typewriting group (n = 60). WJ-WID: Woodcock-Johnson word identification. WJ-WA: Woodcock-Johnson word attack.

	1	2	3	4	5	6	7	8	9
1 English Dictation	—								
2 English Word Reading	0.745***	—							
3 WJ-WID	0.750***	0.872***	—						
4 WJ-WA	0.639***	0.779***	0.723***	—					
5 Orthographic knowledge	0.746***	0.710***	0.757***	0.552***	—				
6 Phonological awareness	0.660***	0.555***	0.589***	0.595***	0.561***	—			
7 English handwriting time	−0.057	0.078	−0.009	0.033	0.113	0.050	—		
8 English typewriting time	0.433***	0.401***	0.303*	0.222^ǂ^	0.324*	0.206	0.295*	—	
9 English typewriting speed	0.631***	0.538***	0.464***	0.492***	0.535***	0.448***	0.031	0.425***	—
10 HKDSE English	0.759***	0.667***	0.623***	0.571***	0.698***	0.562***	0.049	0.165	0.489***

^ǂ^p < 0.1. ^*^p < 0.05. ^**^p < 0.005. ^***^p < 0.001.

**Table 9:** Pearson correlation coefficients between various behavioral measures in English for the pronunciation-based typewriting group (n = 80). WJ-WID: Woodcock-Johnson word identification. WJ-WA: Woodcock-Johnson word attack.

	1	2	3	4	5	6	7	8	9
1 English Dictation	—								
2 English Word Reading	0.784***	—							
3 WJ-WID	0.685***	0.823***	—						
4 WJ-WA	0.665***	0.691***	0.646***	—					
5 Orthographic knowledge	0.709***	0.660***	0.546***	0.447***	—				
6 Phonological awareness	0.389***	0.507***	0.410***	0.446***	0.453***	—			
7 English handwriting time	−0.051	−0.014	−0.070	−0.128	0.073	−0.022	—		
8 English typewriting time	0.144	0.143	0.015	0.054	0.257*	0.084	0.289*	—	
9 English typewriting speed	0.562***	0.448***	0.380***	0.325**	0.458***	0.185	−0.117	0.277*	—
10 HKDSE English	0.613***	0.580***	0.491***	0.525***	0.511***	0.335**	−0.036	0.057	0.401***

^ǂ^p < 0.1. *p < 0.05. **p < 0.005. ***p < 0.001.

Results of fixed-order hierarchical multiple regressions, with the effects of age, HKDSE English, and English handwriting time being statistically controlled, revealed that, for all participants, orthographic knowledge, phonological awareness, time spent on typewriting and typewriting speed are important predictors of English word reading [ΔR^2^ = 0.15, ΔF(1,135) = 42.98, p < 0.001, β = 0.481; ΔR^2^ = 0.10, ΔF(1,135) = 26.33, p < 0.001, β = 0.363; ΔR^2^ = 0.05, ΔF(1,135) = 11.25, p < 0.001, β = 0.233; and ΔR^2^ = 0.05, ΔF(1,135) = 11.52, p < 0.001, β = 0.246, respectively] and dictation [ΔR^2^ = 0.18, ΔF(1,135) = 63.82, p < 0.001, β = 0.519; ΔR^2^ = 0.06, ΔF(1,135) = 15.97, p < 0.001, β = 0.273; ΔR^2^ = 0.05, ΔF(1,135) = 14.00, p < 0.001, β = 0.240; and ΔR^2^ = 0.09, ΔF(1,135) = 27.92, p < 0.001, β = 0.340, respectively] (see Table 10 for the regression table). Similar result patterns were observed for the orthographic group and the pronunciation group, except that time spent on typewriting did not account for significant amount of variance in English word reading [ΔR^2^ = 0.016, ΔF(1,75) = 1.81, p = 0.182, β = 0.133] and English dictation [ΔR^2^ = 0.015, ΔF(1,75) = 1.84, p = 0.179, β = 0.130] for the pronunciation group. When English typewriting speed was the criterion variable, English orthographic knowledge and time spent on English typewriting accounted for significant portion of variance in English typewriting speed [ΔR^2^ = 0.07, ΔF(1,135) = 13.47, p < 0.0005, β = 0.336 and ΔR^2^ = 0.11, ΔF(1,135) = 20.83, p < 0.0005, β = 0.349, respectively]. Phonological awareness did not account for significant portion of variance in English typewriting speed [ΔR^2^ = 0.01, ΔF(1,135) = 1.27, p = 0.262]. The results suggest that literacy skills, orthographic knowledge and typewriting proficiency are interrelated in English. Better orthographic knowledge correlates positively with processes that recruit this cognitive skill, namely reading, spelling and typewriting. This provides cross-linguistic evidence to the hypothesis that typewriting methods that recruit similar cognitive processes crucial for successful reading acquisition will preserve or promote literacy skills of fluent readers. Conversely, good spelling skills, but not good phonological awareness, is crucial for good typewriting speed in English.

**Table 10:** Summary of hierarchical multiple regressions that tested the predictive power of English orthographic knowledge, phonological awareness and English typewriting measures on English reading, dictation and typewriting speed after controlling for differences in age, HKDSE English and English handwriting time

Step	Variable	All participants (n = 140)											
		English Word Reading				English Dictation				English Typewriting Speed			
		ΔR^2^		β		ΔR^2^		β		ΔR^2^		β	
1	Age	0.012				0.013				0.001			
2	HKDSE English	0.363^***^				0.441^***^				0.186^***^			
3	Handwriting time	0.002				0.001				0.005			
4	Typewriting speed	0.049^***^		0.246^***^		0.093^***^		0.340^***^		—		—	
4	Typewriting time	0.048^***^		0.233^***^		0.051^***^		0.240^***^		0.108^***^		0.349^***^	
4	Orthographic knowledge	0.150^***^		0.481^***^		0.175^***^		0.519^***^		0.073^***^		0.336^***^	
4	Phonological awareness	0.090^***^		0.334^***^		0.059^***^		0.271^***^		0.010		0.114	
**Step**	**Variable**	**Orthographic-based typewriting group (n = 60)**						**Pronunciation-based typewriting group (n = 80)**					
		**English Word Reading**		**English Dictation**		**English Typewriting Speed**		**English Word Reading**		**English Dictation**		**English Typewriting Speed**	
		**ΔR** ^**2**^	**β**	**ΔR** ^**2**^	**β**	**ΔR** ^**2**^	**β**	**ΔR** ^**2**^	**β**	**ΔR** ^**2**^	**β**	**ΔR** ^**2**^	**β**
1	Age	0.049^ǂ^		0.037		0.009		0.002		0.001		0.002	
2	HKDSE English	0.411^***^		0.546^***^		0.231^***^		0.338^***^		0.374^***^		0.160^***^	
3	Handwriting time	0.003		0.008		0.000		0.000		0.001		0.010	
4	Typewriting speed	0.057^*^	0.274^***^	0.088^***^	0.340^***^	—	—	0.057^*^	0.263^*^	0.118^***^	0.378^***^	—	—
4	Typewriting time	0.080^**^	0.302**	0.122^***^	0.373^***^	0.131^***^	0.386^***^	0.016	0.133	0.015	0.130	0.089^**^	0.317**
4	Orthographic knowledge	0.108^***^	0.464^***^	0.095^***^	0.435 ^***^	0.073^*^	0.381^*^	0.179^***^	0.495^***^	0.217^***^	0.546^***^	0.095**	0.360**
4	Phonological awareness	0.039*	0.242*	0.076^***^	0.339^***^	0.043^ǂ^	0.256^ǂ^	0.111^***^	0.354^***^	0.038^*^	0.207^*^	0.003	0.055

^ǂ^p < 0.10 ^*^p < 0.05 ^**^p < 0.005 ^***^p < 0.001.

Interestingly, results of one-way ANCOVA, where the age, Chinese and English HKDSE performance, Chinese and English typewriting speed and time and phonological awareness were statistically controlled, showed that the two groups did not differ in any English reading-related measures, but they differed significantly in Chinese character reading [adjusted means for the orthographic and pronunciation groups are 270.88 and 265.06, respectively; *F*(1,130) = 4.82, *p* < 0.05, η^2^ = 0.04] and Chinese dictation [adjusted means for the orthographic and pronunciation groups are 67.29 and 61.48, respectively *F*(1,130) = 6.49, *p* < 0.05, η^2^ = 0.05]. So typewriting experience in one language affects mostly the literacy performance in the same language without cross-language transfer.

## General Discussion

Results of the present research have demonstrated that orthographic-based and pronunciation-based inputting skills bear different relationships with Chinese reading and writing measures. Orthographic-based typewriting time and speed correlated positively with dictation and marginally with reading performance, whereas pronunciation-based typewriting time did not correlate with reading performance. Orthographic knowledge correlated significantly with character reading and dictation in both groups, and typewriting speed was contingent on orthographic knowledge for orthographic-based typists but not the pronunciation-based typists. These findings suggest that successful Chinese reading and writing require good knowledge of orthographic form of characters, and orthographic-based input methods, such as Cangjie, Quick and Stroke, may promote and preserve this knowledge. These input methods entail cognitive skills that are crucial for dictation, such as visual imagery and analysis of the orthographic structure of characters and conversion of character components or strokes into designated keyboard codes. Pronunciation-based typewriting, on the other hand, requires activations of phonological codes of characters and conversions of these codes into Pinyin symbols. As homophones abound in Chinese, inputting the Pinyin string of a syllable will retrieve a list of characters that share the same syllable and a typist needs to choose the right character from among the choices. A favorable feature of the pinyin input method is that it allows users to type just the initials of the pinyin code of multisyllabic words to speed up the typing process. Indeed, our results have shown that the pronunciation-based typewriting group has significantly faster overall typing speed than the orthographic-based typewriting group (141.41 vs. 109.14 words). As was reported by Chen and Chuang^40^, phonology-based (i.e., Zhuyin) users had better phonological sensitivity but poorer orthographic awareness and tended to make more phonological-related typing errors, whereas orthographic-based (i.e., Canjie) users showed the opposite pattern of results. A similar trend was shown in our study, with the pronunciation-based group having slightly better phonological awareness than the orthographic-based group (15.07 vs. 13.83), though the difference was not statistically significant (see Table 6). Their findings, together with ours, suggest that the pronunciation-based input methods may promote phonological awareness and help disadvantaged readers with poor reading, writing, and/or visual memory of characters to write efficiently and promote their sound-to-print connections. However, as orthographic awareness is not promoted using this method, the positive side of pronunciation-based typing will be offset by this negative side, and children using this input method will have poor reading and writing skills, as is demonstrated by Tan *et al*.’s^42^ findings with primary school children and our study with proficient adult readers.

Past studies have shown that multiple cognitive skills are found to predict Chinese reading development. They include phonological awareness^41,48^, visual processing skills^41,49,50^, orthographic processing skills^41,51^, morphological awareness^52–55^, speeded naming skills^37,51^, and hand copying skills^37^. These findings suggest that multiple learning strategies are used in learning to read Chinese, but the most important strategy has yet to be identified. Results of the current study may imply that visual-orthographic skills play a more important role than phonological skills in Chinese reading and writing, at least for our mature readers to maintain their literacy skills. While it is a bit surprising that pronunciation-based input methods did not correlate with writing/dictation skills, possibly because of the high proficiency in Chinese literacy of the participants that makes the negative impact less detectable, increasing use of these methods did negatively impact reading accuracy in Chinese fluent readers.

In the alphabetic literature, handwriting skills have not been found to predict reading outcomes^7–9^, and numerous studies have reported a positive relation between typewriting and literacy development in normal and disabled children as well as adults^32–36,56–61^. Our findings on ESL learners have also shown that typing-related measures positively correlate with reading and dictation skills in English, suggesting that reduced handwriting practice and increasing use of typewriting will not detrimentally affect literacy development in English.

Contrary to alphabetic language, handwriting and reading are closely related in Chinese. Tan *et al*.^37^ showed that children’s reading ability is strongly associated with their handwriting ability and postulated that handwriting strengthens orthographic awareness and establishes motor programs that lead to the formation of long-term motor memories of Chinese characters. Similarly, in a second language training study among American adults, Guan *et al*.^39^ reported that handwriting training helps Chinese character recognition by refining the visual-spatial information needed for character recognition and adding a sensorimotor memory that accompanied handwriting. In a recent fMRI study, Cao and Perfetti^62^ argued that reading-writing connection is stronger in Chinese than English reading, and American adults who learned Chinese characters using handwriting practice have stronger brain activity in the left middle frontal gyrus (a brain region found to be important for Chinese reading) than those who learned without handwriting practice. Reduced handwriting thus may hinder reading and writing development in Chinese. In the digital era, it is quite unlikely to prevent children from using digital devices for information acquisition and literacy learning. It is thus important to use a keyboard input method that resembles the cognitive processes involved in handwriting. Our findings suggest that orthographic-based input methods are more preferable to pronunciation-based input methods, although the latter are easier to acquire and allow for faster typing speed. For young or disabled learners with motor immaturity or difficulties to read and write, they may find it attractive to read and write using electronic devices and may find the pronunciation-based typewriting method easier and more efficient to use. The easiness of pronunciation-based typewriting may offset the negative impacts that it may bring and motivate those who otherwise are reluctant or even avoid writing at all to typewrite. More research is needed to examine the impact of increasingly used keyboarding techniques on children’s reading and writing skills, and to design the best pedagogical approaches in teaching keyboarding techniques to developing readers.
